# *Rhodobacter sphaeroides* as a model to study the ecotoxicity of 1-alkyl-3-methylimidazolium bromide

**DOI:** 10.3389/fmolb.2023.1106832

**Published:** 2023-01-30

**Authors:** Xiao-Lin Liu, Ming-Qing Chen, Yang-Lin Jiang, Rong-Yao Gao, Ze-Jun Wang, Peng Wang

**Affiliations:** Department of Chemistry, Renmin University of China, Beijing, China

**Keywords:** *Rhodobacter sphaeroides*, ionic liquids, cytotoxicity, membrane integrity, spectral probe

## Abstract

The purple non-sulfur bacterium *Rhodobacter sphaeroides* was selected as a biological model to investigate its response to the toxicity of 1-alkyl-3-methylimidazolium bromide ([C_n_mim]Br), a type of ionic liquid (IL), with different alkyl chain lengths (*n* describes the number of carbon atoms in the alkyl chain). The inhibition of bacterial growth by [C_n_mim]Br was positively correlated with *n*. Morphological characterization revealed that [C_n_mim]Br caused cell membrane perforation. The signal amplitude of the electrochromic absorption band shift of endogenous carotenoids showed a negatively linear correlation with *n*, and the amplitude of the blue-shift of the B850 band in light-harvesting complex 2 showed a positively linear correlation with *n*. Furthermore, an increase in blocked ATP synthesis and increase in antioxidant enzyme activity were observed in chromatophores treated with ILs containing longer alkyl chains. In summary, the purple bacterium can be developed as a model to monitor ecotoxicity and examine the mechanism of IL toxicity.

## 1 Introduction

Ionic liquids (ILs), having the properties of negligible vapor pressure, non-flammability, good stability, high ionic conductivity, and good designability, are considered a perfect substitute for organic solvents in many applications (Maase and Massonne, 2005; Dong et al., 2017; Wang et al., 2017; Joshi and Adhikari, 2019; Greer et al., 2020; Beil et al., 2021). Over the past decade, the potentially (eco)-toxicological hazards of ILs have drawn great attention (Jastorff et al., 2003; Bubalo et al., 2014; Bubalo et al., 2017; Cho et al., 2021). Different model organisms from various ecological niches—including bacteria, fungi, algae, nematodes, water fleas, invertebrates, fish, plants, and mammalian cell culture—have been used to evaluate the toxicity of ILs (Pretti et al., 2006; Ventura et al., 2010; Ghanem et al., 2015; Pernak et al., 2015; Zhu et al., 2016; Biczak et al., 2017; Chen et al., 2020; Chu et al., 2022; Vega et al., 2022). Of these, microorganisms have been used extensively as model indicators to study the environmental toxicity of ILs due to their wide distribution in nature, short reproduction time, easy cultivation and observation, and ecological and industrial relevance (Sumampouw and Risjani, 2014; Zhang et al., 2022). Previous investigations mainly focused on the relationship between the chemical structure of ILs and their toxicity. Studies have indicated that the inhibition of bacterial growth by ILs is due to their affinity and disruptive effect of ILs on the cell membrane and cell wall (Docherty and Kulpa, 2005; Cornmell et al., 2008; Busetti et al., 2010; Ventura et al., 2013; Ghanem et al., 2015; Zheng et al., 2016; Cook et al., 2019; Pałkowski et al., 2022). The cationic structure of ILs is key to their toxicity because it allows ILs to be easily adsorbed and enriched in the negatively charged cell walls of bacteria. Notably, the lipophilicity of the cationic groups on ILs is positively correlated with the degree of damage to the phospholipid bilayer, which causes the narcotic effects. By contrast, ILs anions are considered less toxic (Wang et al., 2011).

Purple non-sulfur bacteria (PNSB), a Gram-negative bacterium, is the main producer in aquatic ecosystems; PNSB occupies important ecological niches in the biosphere and is widely spread in nature, even in some extreme environments (Bryant and Frigaard, 2006; Madigan and Jung, 2009). *Rhodobacter sphaeroides*, a model PNSB*,* has been used for monitoring water pollution and remediating water bodies due to its sensitivity to contaminants, including heavy metal ions and pesticides (Balsalobre et al., 1993; Chalam et al., 1996; Kis et al., 2015). However, studies examining the toxicity and the corresponding mechanism of ILs to *R*. *sphaeroides* or other PNSB are limited*.*

**FIGURE 1:**
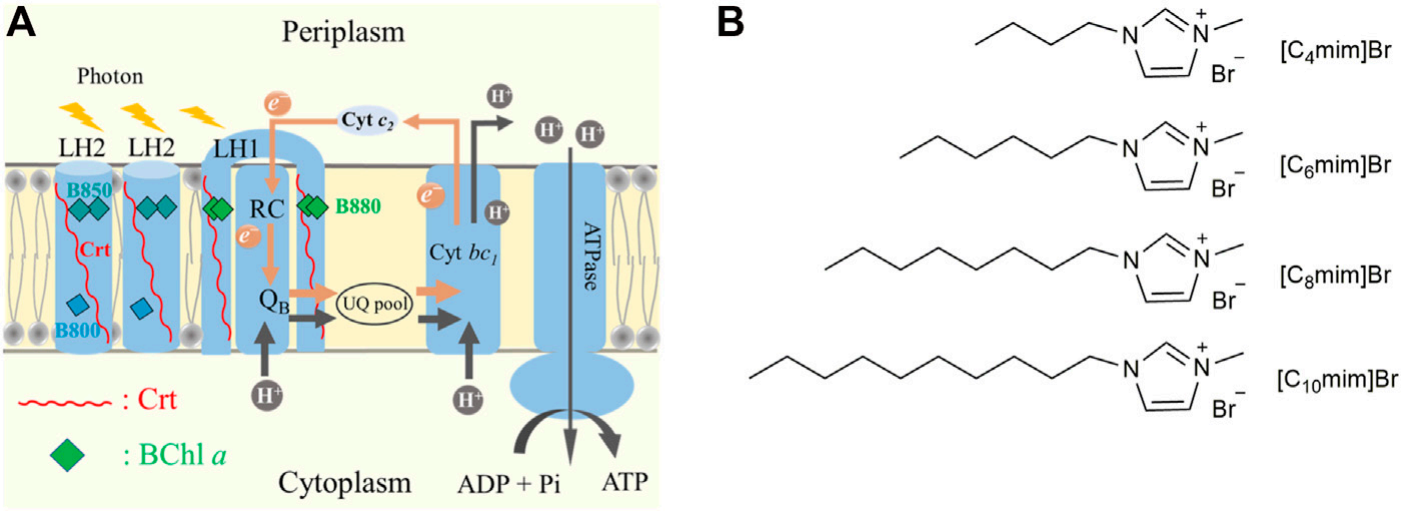
**(A)** A cartoon of the chromatophore of *Rhodobacter sphaeroides.* Proton-coupled electron transfer (PCET) is depicted in gray and brown arrows. Carotenoid (Crt) is shown as a red line and bacteriochlorophyll (BChl) *a* is represented with squares of different colors. Cyan and dark cyan represented B800 and B850 in LH2, respectively. Green represents B880 in LH1-RC. **(B)** Chemical structures of [C_4_mim]Br, [C_6_mim]Br, [C_8_mim]Br, and [C_10_mim]Br.

In PNSB, the chromatophore performs the main function of photoreaction, i.e., photosynthetic phosphorylation. It is mainly composed of two types of pigment–protein complexes, namely light-harvesting complex 2 (LH2) and light-harvesting 1–reaction center supercomplex (LH1-RC). Other important functional proteins include cytochrome *bc*1 and ATPase (Figure 1A) (Cogdell et al., 2006). In LH2 and LH1-RC, the non-covalently binding bacteriochlorophyll (BChl) *a* and carotenoid (Crt) are ideal endogenous spectral probes to detect changes in the lipid microenvironment and the interaction between membrane proteins. For example, the Q_y_ absorption band of BChl *a* in LH2 was shown to be sensitive to its aggregation status in the membrane (Westerhuis et al., 1998; Kangur et al., 2008; Sumino et al., 2013), and the S_0_→S_2_ absorption band of Crt exhibited band-shift in response to change in transmembrane potential difference (Δ*Ψ*) (Jackson et al., 1969; Dijkema et al., 1980; Holmes et al., 1980). Using the latter method, Malferrari et al. (2015) investigated the effect of ILs on the chromatophores of purple bacteria and found that cation structure and alkyl chain length of the imidazolyl cation regulated the permeability of the chromatophore membrane. In our previous study, this method was used to evaluate the effects of detergents on membrane permeability (Zhou et al., 2018).

Imidazolium bromide, as one of the most extensively studied ILs due to its great potential to be used in industries (Chu et al., 2022), has been reported with toxicity to various bio-organisms. Very recent study claimed that the alkyl chain length of imidazolium cation was correlated with its toxicity. (Gonçalves et al., 2021) To further explore the intrinsic mechanism of this type of ILs on prokaryotes, in this study, the effects of 1-alkyl-3-methylimidazolium bromide ([C_n_mim]Br), containing different alkyl chain lengths (*n* = 4, 6, 8, and 10), were evaluated on the growth, physiological function*,* and membrane integrity of *R*. *sphaeroides*. The microstructure of IL-treated bacterial cells was characterized by scanning electron microscopy (SEM). The absorption spectra of endogenous probes Crt and BChl *a* were used to evaluate the effect of ILs on the membrane permeability and aggregation status of membrane proteins. The activities of ATPase and antioxidation enzymes were measured to examine the physiological effects of ILs. Herein, the structure-effect relationship of [C_n_mim]Br on *R*. *sphaeroides* are systematically discussed.

## 2 Materials and methods

### 2.1 Materials

Four [C_n_mim]Br ILs, namely 1-butyl-3-methylimidazolium bromide ([C_4_mim]Br), 1-hexyl-3-methylimidazolium bromide ([C_6_mim]Br), 1-octyl-3-methylimidazolium bromide ([C_8_mim]Br), and 1-decyl-3-methylimidazolium bromide ([C_10_mim]Br) (Figure 1B), were purchased from InnoChem Science & Technology Co., Ltd. (Beijing, China). Kits for detecting inorganic phosphate, superoxide dismutase (SOD) activity, and catalase (CAT) activity were purchased from Solarbio Science & Technology Co., Ltd. (Beijing, China). *R*. *sphaeroides* was derived from the frozen samples in the laboratory. Other reagents were purchased from Sinopharm Chemical Reagent Co., Ltd. (Shanghai, China). All reagents used were analytical grade.

### 2.2 Bacterial cultivation and chromatophore preparation

Bacterial cultivation and chromatophore preparation have been described (Hunter and Turner, 1988). Briefly, frozen *R*. *sphaeroides* were taken out of−80°C, inoculated into M22 + liquid medium, and anaerobically cultivated under a 60-W tungsten lamp for 3–5 days after dark adaptation for 12 h. Bacterial cells were harvested by centrifugation and resuspended in 20 mM Tris-HCl (pH 8.0). The chromatophore was collected by centrifugation (254 000 × *g*, 4°C, 1 h) after the cells were disrupted by ultrasonication. The surface layer of the sediment was gently brushed and homogenized with 20 mM Tris-HCl (pH 8.0), and the chromatophore was diluted to a final concentration of OD_850_ = 50 cm^−1^.

### 2.3 Toxicity of ILs on PNSB

Bacteria were suspended in M22 + medium with 10^5^–10^6^ colony-forming units per mL (CFU·mL^-1^) and exposed to 0–128 mM of ILs. After 4 h of dark adaptation, the bacteria were cultured under illumination. The optical density of the bacterial solution at 660 nm (OD660) was recorded after incubation for 96 h. The half maximal inhibitory concentration (IC_50_) of ILs to PNSB was fitted according to survival rate-concentration curves using Origin 2021 software.

### 2.4 Morphological evaluation of bacteria by SEM

Samples for SEM were prepared as described (Xiao et al., 2017). Briefly, *R*. *sphaeroides* was incubated with 5 mM ILs for 4 h, collected by centrifugation, and washed three times with PBS. Bacterial cells were fixed with 2.5% glutaraldehyde, dehydrated with a 20%–100% ethanol gradient for 0.5 h each time, and air-dried. The surface morphology of bacteria was imaged using SEM (S-4800, Hitachi, Tokyo, and Japan).

### 2.5 Membrane integrity of *R*. *sphaeroides*

The exogenous fluorescent probe propidium iodide (PI) was used to evaluate cell membrane integrity. Live bacteria were incubated in 10 mM PBS (pH 7.4), 20 μM PI, and 5 mM ILs for 30 min in the dark at 25°C. The unabsorbed dye was removed by washing and centrifugation (15,000 × *g*, 3 min) three times. The bacterial cells were resuspended in PBS buffer. PI fluorescence was determined by the FLS 980 fluorescence spectrometer (Edinburgh Instruments, Edinburgh, United Kingdom) at *λ*_ex_ = 538 nm and *λ*_pr_ = 617 nm.

### 2.6 UV-Vis absorption spectrum of the chromatophore exposed to [C_n_mim]Br

The Q_y_ absorption bands of BChl *a* in LHs can be used as a spectral indicator to evaluate changes in the supercomplex structure of the cell membrane. The UV-Vis NIR absorption spectrum of the chromatophore incubated with 5 mM [C_n_mim] Br for 4 h under dark was recorded using the Cary-60 UV-Vis spectrophotometer (Agilent, California, United States).

### 2.7 The electrochromic absorption band shift spectra of Crt

The measurement of the electrochromic absorption band shift of Crt is described (Zhou et al., 2018). Briefly, the chromatophore from *R*. *sphaeroides* was diluted to a final concentration of OD_850_ = 0.55 cm^−1^ in 10 mM MOPS (pH 7.3) buffer containing 20 mM ascorbate sodium (Vc-Na), 2 μM phenazine methyl sulfate (PMS), and 5 mM IL. The test system was based on the Cary-60 UV-Vis absorption spectrometer and modified using a homemade accessory (Supplementary Figure S1). The absorption spectra of the chromatophore, measured under excitation “without” or “with” an additional continuous actinic laser, were termed “Dark” and “Light,” respectively. The electrochromic absorption band shift was characterized by the differential spectra of “Light”-minus-“Dark.” All tests were repeated five times.

### 2.8 ATP synthesis test

The ATP synthesis assays were performed as described (Kim et al., 2017). Briefly, the chromatophore isolated from *R*. *sphaeroides* was diluted to a final concentration of OD_850_ = 5 cm^−1^ in working buffer (20 mM Tris-HCl (pH 8.0), 20 mM Vc-Na, 2 μM PMS, 5 mM K_2_HPO_4_, 5 mM ADP, and 10 mM MgSO_4_). [C_n_mim]Br was added to the working solution at a final concentration of 5 mM. ATP synthesis was performed under illumination with a xenon lamp (15 W/m^2^) at 30°C for 15 min, and then terminated by adding 4% trichloroacetic acid. A tissue inorganic phosphorus content detection kit was used to measure the consumption of inorganic phosphate in the reaction system. The amount of ATP generated was calculated based on ADP + Pi → ATP under illumination. The reference experiment was performed by adding 0.2 mM *N, N′*-dicyclohexylcarbodiimide (DCCD), an inhibitor of F_0_F_1_-ATP synthase (Zürrer et al., 1983). ATPase activity was calculated compared with the control group under illumination.

### 2.9 Activity of antioxidant enzymes

*R*. *sphaeroides* was incubated with 5 mM [C_n_mim]Br for 4 h under light. The bacterial cells were collected after centrifugation and lysed ultrasonically for 15 min. The concentration of total soluble cellular protein in suspension was determined using the Bradford method (Bradford, 1976). Commercial kits were used to determine the activity of SOD and CAT.

## 3 Results

### 3.1 Toxicity of ILs on *R*. *sphaeroides*

**FIGURE 2:**
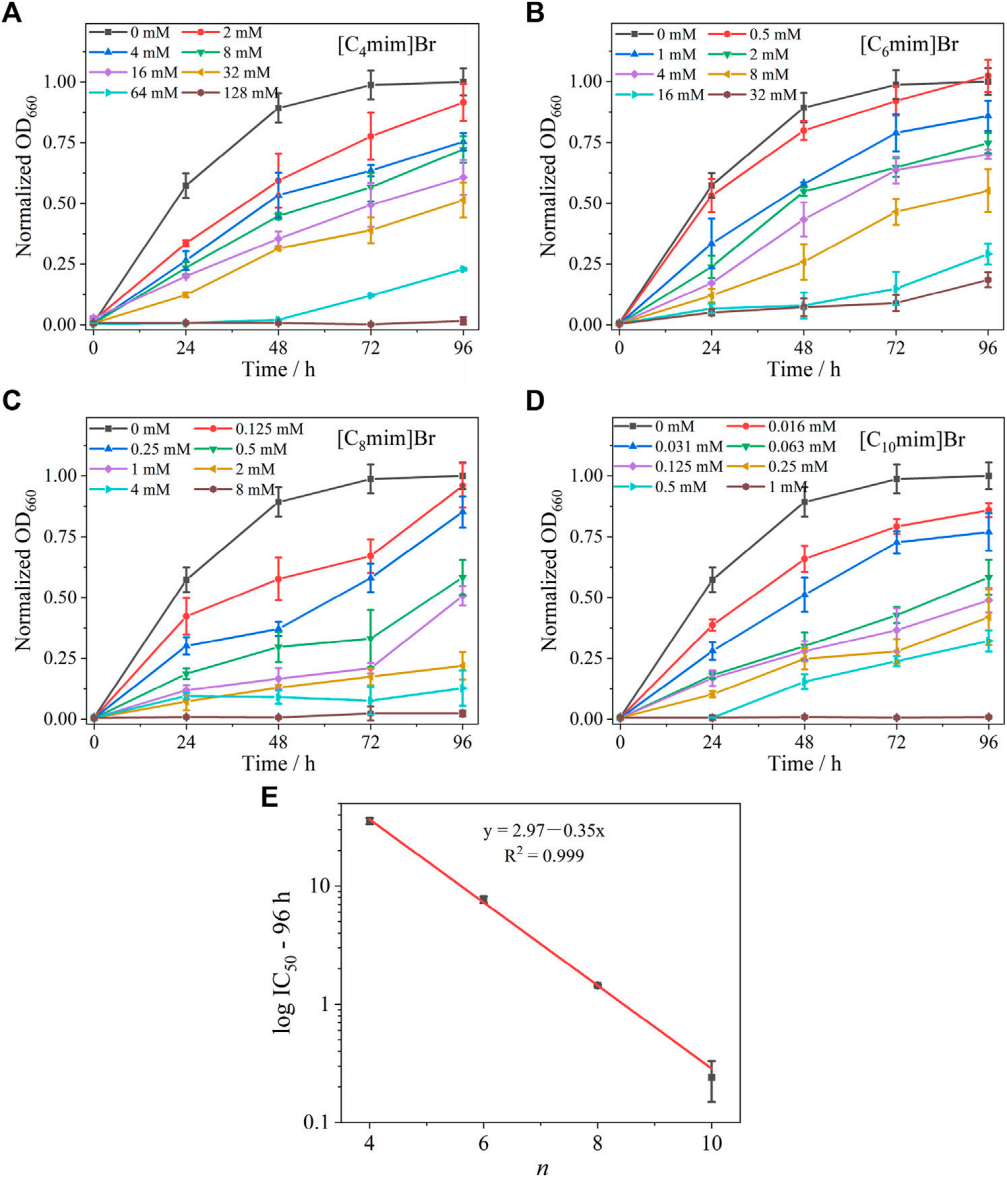
Growth curves of *Rhodobacter sphaeroides* incubated with various concentrations of ILs with different alkyl chain lengths for 96 h, **(A)** [C_4_mim]Br, **(B)** [C_6_mim]Br, **(C)** [C_8_mim]Br, and **(D)** [C_10_mim]Br. Each test had three biological replicates. Standard deviations (SD) are shown as error bars. **(E)** Relationship between log IC_50_ of [C_n_mim]Br at 96 h and alkyl chain length (*n*) of the imidazolium cation.

The effect of C_n_mim]Br ILs on the growth of *R*. *sphaeroides* is shown in Figures 2A–D. All four ILs exhibited a significant inhibitory effect on bacterial growth. In the absence of ILs, bacteria grew to the logarithmic growth phase within 24 h and reached the stationary phase after 72 h. After the addition of ILs, taking [C_4_mim]Br as an example (Figure 2A), bacterial growth was retarded at low concentrations (2 mM). Growth inhibition became more pronounced with increasing IL concentration, and the bacteria ceased to grow in the presence of 128 mM [C_4_mim]Br. ILs with different alkyl chain lengths showed the same trend—a positive correlation between concentration and inhibition rate. Comparing the effect of alkyl chain length on bacterial growth revealed that the longer the carbon chain, the more significant the inhibition. Bacterial growth exposed to 2 mM [C_n_mim]^+^ is shown in Supplementary Figure S2. The IC_50_ values of [C_4_mim]Br, [C_6_mim]Br, [C_8_mim]Br, and [C_10_mim]Br at 96 h were 35.68 ± 2.21, 8.25 ± 0.49, 1.08 ± 0.03, and 0.25 ± 0.09 mM, respectively (Table 1), which are comparable to those reported for other Gram-negative bacteria (Ghanem et al., 2015). An excellent linear positive correlation between the logarithmic concentration of IC_50_ and the corresponding alkyl chain length of IL was observed (Figure 2E). Given that the log*P* (oil–water partition coefficient) of the four ILs gradually increased from [C_4_mim]Br to [C_10_mim]Br (Supplementary Table S1) (Fan et al., 2016), the toxicity of ILs to *R*. *sphaeroides* was positively correlated with their hydrophobicity. This result was consistent with others on IL toxicity in bacteria (Pernak et al., 2007; Łuczak et al., 2010).

**TABLE 1:** The IC_50_ of [C_n_mim]Br on *R. sphaeroides*.

	IC_50_-96 h
[C_4_mim]Br	35.68 ± 2.21
[C_6_mim]Br	8.25 ± 0.49
[C_8_mim]Br	1.08 ± 0.03
[C_10_mim]Br	0.25 ± 0.09

### 3.2 Membrane integrity analysis by SEM and PI fluorescent probes

**FIGURE 3:**
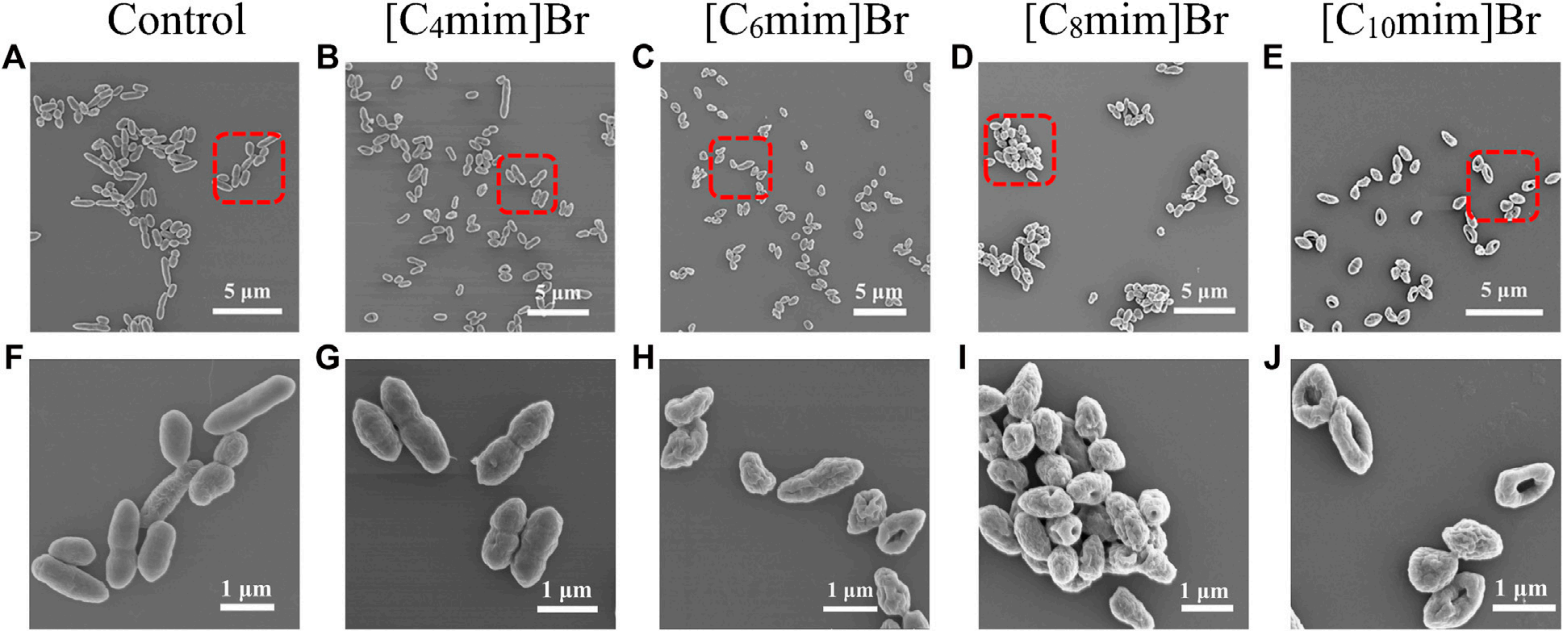
Scanning electron micrographs of *Rhodobacter sphaeroides* after incubation without **(A**, **F)** and with 5 mM [C_n_mim]Br **(B–E)**, **(G–J)** in M22 + medium for 12 h in light **(B–E)**. The corresponding detailed images **(G–J)** represent [C_4_mim]Br, [C_6_mim]Br, [C_8_mim]Br, and [C_10_mim]Br, respectively.

The morphology of *R*. *sphaeroides* was examined by SEM (Figure 3). The untreated bacterial cells were typically long and rod-shaped, approximately 1.5 × 0.5 μm in size, with an intact envelope and smooth surface and no adhesion (Figure 3A). After IL treatment (Figures 3B–E), the bacteria gradually lost their rod-like shape and shrank in size to a minimum of 0.9 μm × 0.5 μm, with a rough and concave surface. In scaled-up figures (Figures 3G–J), pores and cavities are visible on the surfaces of bacteria treated with longer alkyl chain ILs, such as [C_10_mim]Br (Figure 3J). These results clearly indicate that ILs damaged the bacterial cell wall and cell membrane. This structural damage inhibited cell growth. The degree of morphological change is positively dependent on the alkyl chain length of [C_n_mim]Br, consistent with other reports (Jeong et al., 2012; Mehmood et al., 2015).

PI is used to stain nuclei. PI exhibits enhanced fluorescence after intercalating with DNA. As a water-soluble dye, PI is used as an exogenous probe to evaluate cell membrane integrity because it cannot get through the intact membrane of living cells (Petkovic et al., 2012). The fluorescence intensity of PI up-taken by bacteria that were incubated with 5 mM ILs is shown in Supplementary Figure S3. PI fluorescence increase corresponded with the increase in alkyl chain length. This result is consistent with the results of SEM analysis. Surface potential measurements of bacteria revealed that ILs with longer chains were adsorbed more easily on the surface of the bacterial cell wall, because surface potential increased from−17−13 mV with the increase in alkyl chain length (Supplementary Figure S4).

### 3.3 Endogenous spectral probes on the cytoplasmic membrane of *R*. *sphaeroides* responding to the effect of ILs

**FIGURE 4:**
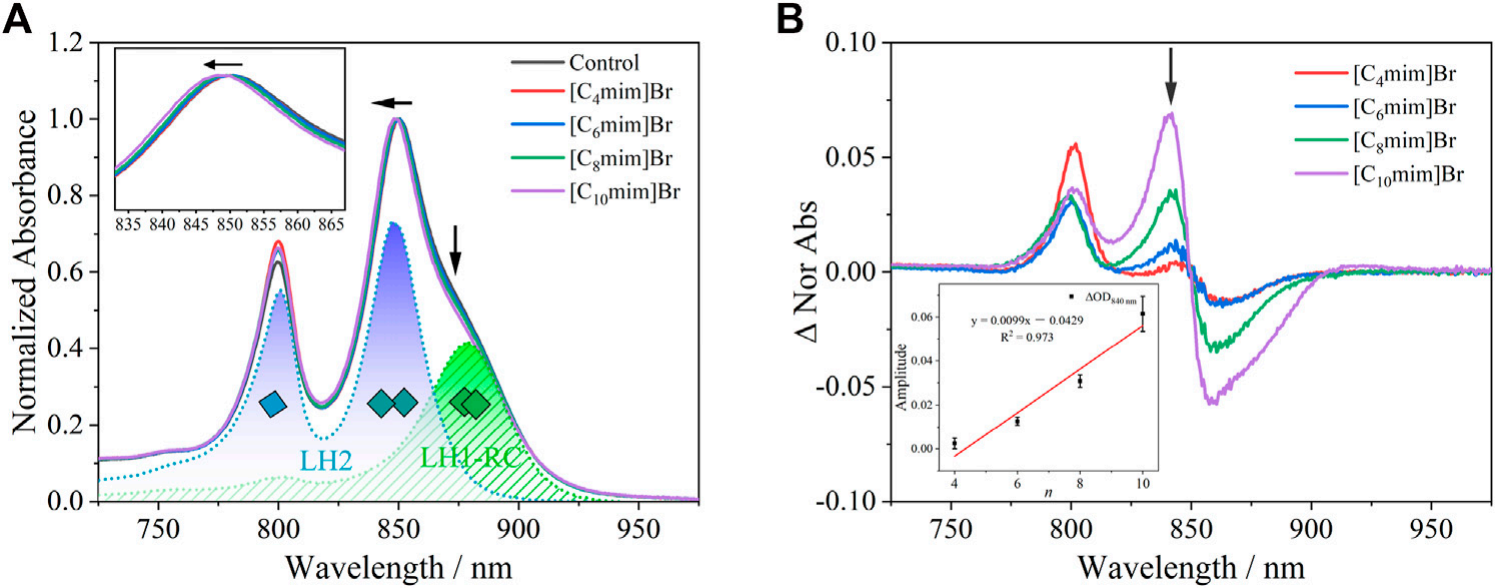
**(A)** UV-Vis absorption spectrum of chromatophore after being exposed to 5 mM [C_n_mim]Br for 4 h. Peaks at 800 and 850 nm were attributed to B800 and B850 in LH2, respectively (blue dot line), and peak at 880 nm was attributed to B880 in LH1 (green dot). The inserted graph shows a blue-shift at B850 in [C_n_mim]Br compared with the control group. **(B)** Differential spectra of the chromatophore between [C_n_mim]Br and control. The inserted graph shows a linear relationship between *n* and the amplitude of ∆_840 nm_.

The baseline-corrected near-infrared region absorption spectrum of the chromatophore, the debris of the cytoplasmic membrane of *R*. *sphaeroides*, exposed to 5 mM IL solution for 4 h is shown in Figure 4A (normalized at 850 nm). The absorption bands near 800 and 850 nm originate from BChl *a* bound to LH2 and the one near 880 nm from BChl *a* bound to LH1-RC (Figure 1A). LH2, as the main light-harvesting complex, forms aggregate in the cell membrane (Westerhuis et al., 1998; Kangur et al., 2008; Sumino et al., 2013). Treatment with ILs, with increasing alkyl chain lengths, increased the blue-shift of the B850 band, accompanied with a decrease in the intensity of B880 (Figure 4A and the corresponding insert graph). This result indicates that IL insertion decreased LH2 aggregation and disrupted the lipid bilayer structure. More significant variation of spectra was seen as differential spectra between the [C_n_mim]Br-treated or untreated control (Figure 4B). The positive peak at 840 nm and the negative valley at 860 nm were generated in pairs due to the blue-shift of B850 bands in treated samples. The amplitude of ΔOD detected at 840 nm was plotted as a positively linear function of *n* (insert graph in Figure 4B). These results imply that the BChl *a* bound to the chromatophore can be used as a quantitative spectral indicator to evaluate the effects of [C_n_mim]Br on PNSB.

In the PNSB chromatophore, Crt is considered a good endogenous intramembrane voltmeter, because its absorption spectra shift responds to the change in membrane potential, i.e., the Stark effect. Thus, Crt has been widely used as a spectral indicator to evaluate the effect of ionophores antibiotics, detergents, and ILs on membrane permeability (Malferrari et al., 2015; Zhou et al., 2018). In this study, an 808-nm continuous laser was used to initiate the photoinduced “proton-coupled electron transfer” (PCET) in the chromatophore (Figure 1A), which resulted in a transmembrane proton gradient (Δμ_H_^+^). Then the electrochromic absorption band shift of Crt was observed due to the transmembrane Δ*Ψ* accompanying Δμ_H_^+^.

**FIGURE 5:**
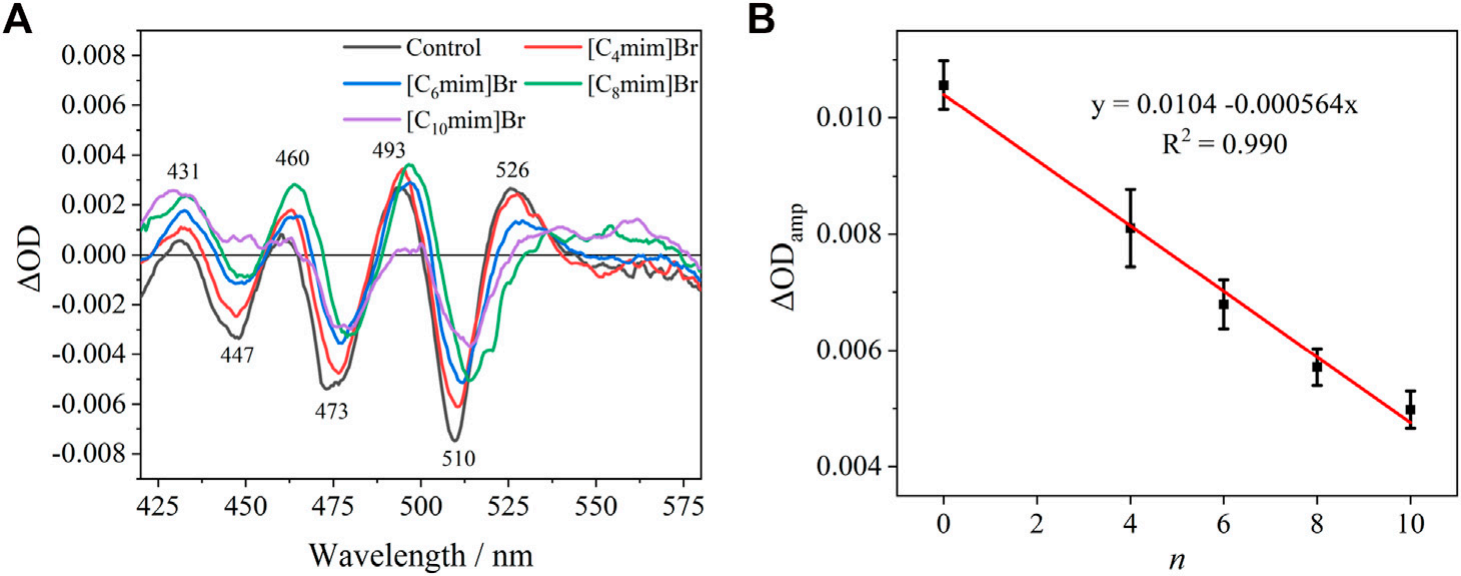
**(A)** “Light”–minus–“dark” differential spectra of carotenoid absorption without or with 5 mM [C_n_mim]Br. **(B)** Relationship between the amplitude of ΔOD_amp_ without or with 5 mM [C_n_mim]Br and the alkyl chain length (*n*) of the imidazolium cation.

The absorption spectra (420–545 nm) of the Crt S_0_→S_2_ transition in the *R*. *sphaeroides* chromatophore under dark and actinic light is shown in Supplementary Figure S5. The Crt absorption bands red-shifted slightly under actinic excitation at 808 nm compared with those under dark. The “light”-minus-“dark” differential spectra, representing the electrochromic absorption band shift of Crt is shown in Figure 5A. The differential spectra had typical oscillating signal peaks at 526, 493, 460, and 431 nm, consistent with a previous report (Zhou et al., 2018). The amplitude of the differential spectra (ΔOD_amp_) decreased linearly with alkyl chain length, i.e., *n* (Figure 5B). To obtain better S/N ratios, ΔOD_amp_ was calculated as ΔOD_526_ − ΔOD_510_. Considering that ILs can decrease the integrity of cell membranes, Δμ_H_^+^ and the accompanying transmembrane Δ*Ψ* would consequently decrease. The corresponding decrease of ΔOD_amp_ upon IL treatment was reasonable. The linearly dependent relationship between ΔOD_amp_ and *n* implied that Crt can be developed as a quantitative and endogenous spectral probe to evaluate the effects of other effectors on biomembranes. Furthermore, this method could be developed for other species of purple bacteria.

### 3.4 Physiological function change upon IL treatment

**FIGURE 6:**
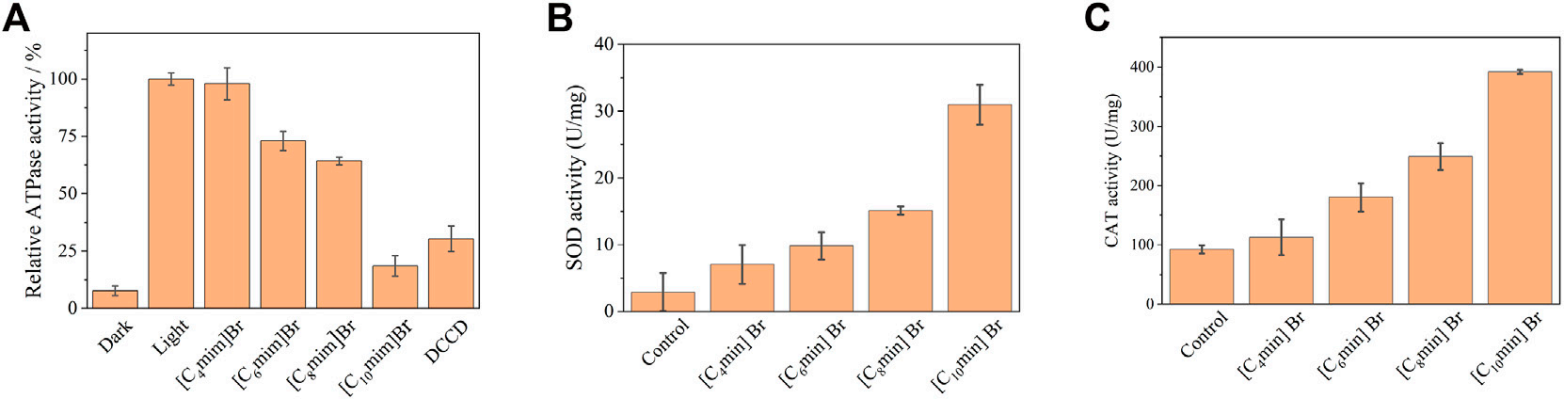
**(A)** Inhibition of chromatophore ATPase activity of *Rhodobacter sphaeroides* with 5 mM [C_n_mim]Br. DCCD, an inhibitor of the F_0_F_1_-ATP synthase covalently binds to the F_0_ subunit. **(B)** SOD and **(C)** CAT activities of *R*. *sphaeroides* treated with [C_n_mim]Br (5 mM). Each test had three biological replicates.

To further investigate the mechanism of IL toxicity in *R*. *sphaeroides*, ATP synthesis and antioxidant enzyme activity were evaluated. Chromatophores isolated from *R*. *sphaeroides* contain the entire photosynthesis unit; therefore, they can utilize light to synthesize ATP. The ATPase activity of chromatophores treated without and with ILs is shown in Figure 6A. Under light, the ATPase activity of the IL-free group was considered 100%. Four ILs exhibited remarkable repressive activity of ATPase in the order of [C_4_mim]Br < [C_6_mim]Br < C_8_mim]Br < [C_10_mim]Br. Notably, for the IL containing the longest alkyl chain ([C_10_mim]Br), ATPase activity drastically decreased to 20%, which is comparable to the effect of the typical proton pump inhibitor DCCD. The result that ATPase activity decreased upon treatment with ILs was consistent with those shown above, where Δμ_H_^+^ decreased upon IL addition. This is because the inability to establish a proton gradient between the cytoplasm and periplasm leads to a blockade of ATP synthesis in the chromatophore.

Antioxidant enzymes are highly expressed in organisms, including PNSB, under environmental stressors, such as light, oxygen, and pollutants, to eliminate damage caused by excess reactive oxygen species production (Zhang et al., 2012; Su et al., 2020). In this study, the activities of intracellular antioxidant enzymes, namely SOD and CAT, were determined in PNSB treated with 5 mM [C_n_mim]Br. The activities of SOD and CAT were 2.90 and 92.31 U/mg without ILs, respectively (Figures 6B, C). However, after IL treatment, the activities of SOD and CAT significantly increased, with a positive correlation with alkyl chain length. Notably for [C_10_mim]Br, the activities of SOD and CAT were the highest, that is 10.7-and 4.3-fold higher than that of the control, respectively. Combined with the results of bacterial morphology analysis, a reasonable conclusion can be drawn that hydrophilic ILs insert and disturb cell membrane structure, leading to membrane perforation, which consequently causes oxidative stress, and eventually, cell death.

## 4 Discussion

In this study, the mechanism of toxicity of four ILs [C_n_mim]Br (*n* = 4, 6, 8, and 10) was evaluated by using *R*. *sphaeroides* as a biological model. The following two issues were addressed:

(1) Various biochemical and physicochemical methods were used to examine the mechanism of toxicity of [C_n_mim]Br on *R*. *sphaeroides*. Because of its cationic properties, [C_n_mim]Br is easily adsorbed on the negatively charged surface of *R*. *sphaeroides*. This can be demonstrated by measuring surface potential. The ILs can pass through the cell wall to attach to the cytoplasmic membrane. With the increase in alkyl chain length, the lipophilicity of ILs increases, facilitating their interaction with the cell membranes, which eventually leads to the perforation of the cell surface. The mechanism of perforation might originate from the strongly electrostatic interaction between the cations of ILs and anions of phospholipids, resulting in the rearrangement of lipid bilayers to form leaky structures on the cell surface (Hartmann et al., 2015; Ciumac et al., 2019; Nikfarjam et al., 2021). SEM analysis and PI staining reveal the structural changes caused by IL treatment on the cell surface, including the cell wall and membrane.

In this study, biochemical and physical changes were monitored using two endogenous spectral probes, i.e., Crt and BChl *a,* that bind to membrane proteins. The Q_y_ absorption bands of BChl *a* embedded in LH2 and LH1-RC could sensitively reflect the structural change of the lipid bilayer. Particularly, the aggregation status of LH2 in native membrane was disturbed by the adsorption of ILs, which generated the blue-shift of the B850 Q_y_ band. By absorption band shift of Crt, the corresponding transmembrane Δ*Ψ* change was confirmed, which eventually were related to the Δμ_H_^+^ due to ILs treatment. Notably, the amplitude of oscillating signals had a linear relationship with alkyl chain length. Thus, this method could be developed to quantitatively evaluate the structure–function relationship of other biomembrane effectors.

The mechanism of IL toxicity was evaluated through enzyme activity tests. Treatment with ILs, decreased ATP synthesis activity and increased antioxidant enzyme activity were observed. The decrease in ATP synthesis activity directly correlated with the decrease in Δμ_H_^+^ caused by IL interference, because the ATPase is driven by a proton gradient. The increase in antioxidant enzyme activity correlated with the increase in SOD and CAT activities, indicating oxidative stress in IL-treated bacteria, which together with the decrease of photosynthetic phosphorylation, eventually led to cell death.

(2) Purple bacteria can be developed as a good biological model to evaluate ecotoxic materials that affect biomembranes (Kis et al., 2015). Purple bacterium is phototrophic and the main producer of aquatic ecosystems (Bryant and Frigaard, 2006; Madigan and Jung, 2009). Although some have been used as to monitor water pollution and remediate water bodies, such as *R. sphaeroides*, they are seldom used as model systems to examine the biochemical and physiological mechanism of toxicity. The findings of this study draw more attention to this aspect. *R. sphaeroides* is a unicellular photosynthetic organism and its cytoplasmic membrane is also its photosynthetic apparatus. The chromatophore can be easily prepared and retains the main functions of light-driven PCET, i.e., photophosphorylation. Furthermore, the embedded Crt and BChl *a* performed well as endogenous spectral probes to reflect the structural changes of the cytomembranes. Thus, developing *R. sphaeroides* as a model for broader applications is warranted.

## 5 Conclusion

In conclusion, the toxicity of [C_n_mim]Br to *R*. *sphaeroides* was firstly correlated with their hydrophobicity. SEM results indicated that longer chain [C_n_mim]Br can damage the bacterial cell wall and cell membrane more easily. Both the PI test and the surface potential assay supported these results. The endogenous pigments, i.e., the bound BChl *a* and Crt, were found to be with the potential as quantitative spectral indicator to evaluate the effects of [C_n_mim]Br on PNSB. This was strengthened by additional tests on ATPase activity and antioxidant enzyme activity. Combined all results, the toxicity of [C_n_mim]Br to PNSB was summarized as that hydrophilic ILs inserted and disturbed cell membrane structure, leading to membrane perforation, which consequently causes oxidative stress, and eventually, cell death.

Our exploration could be developed for other species of purple bacteria, and the purple bacterium can be developed into a model system to evaluate ecotoxicity and examine the mechanism of toxicity of various effectors on biomembranes.

## Data Availability

The original contributions presented in the study are included in the article/Supplementary Material, further inquiries can be directed to the corresponding author.
