# Supplementary material for: Rhodobacter sphaeroides as a model to study the ecotoxicity of 1-alkyl-3-methylimidazolium bromide
Source: Front Mol Biosci. 2023 Jan 30;10:1106832. doi: 10.3389/fmolb.2023.1106832 (PMC9923006; doi:10.3389/fmolb.2023.1106832)
Supplement: Supplementary file 1 [file Table1.DOCX]

Supplementary Material

*Rhodobacter sphaeroides* as a biological model to study the eco-toxicity and its mechanism of 1-alkyl-3-methylimidazolium bromide with various alkyl lengths

Xiao-Lin Liu, Ming-Qing Chen, Yang-Lin Jiang, Rong-Yao Gao, Ze-Jun Wang, Peng Wang^*^

Department of Chemistry, Renmin University of China, Beijing, 100872, People’s Republic of China

* Corresponding author.

E-mail address: [wpeng_chem@ruc.edu.cn](mailto:wpeng_chem@ruc.edu.cn) (P. Wang).


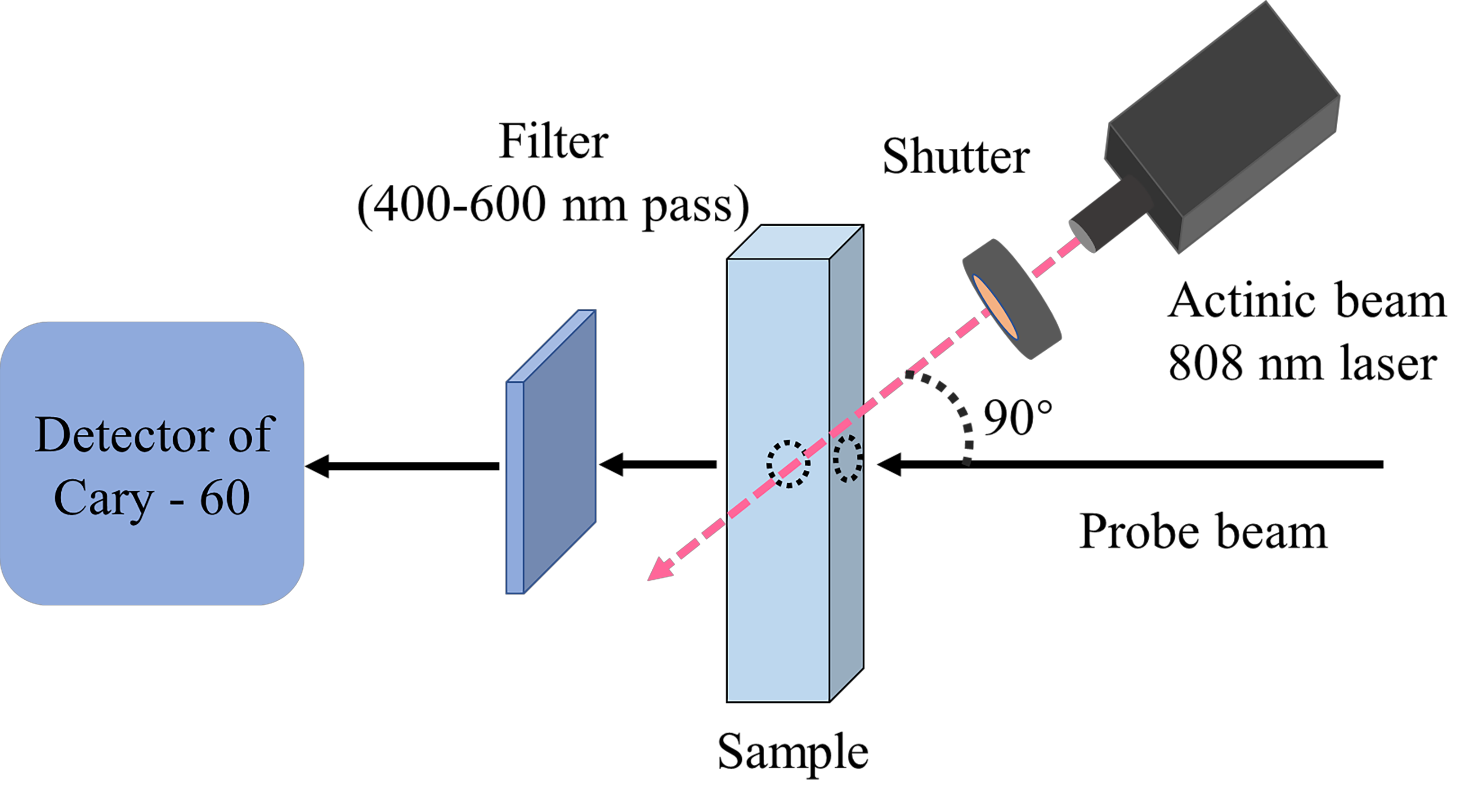


**Supplementary Figure S1** A scheme for homemade accessory based on a Cary-60 UV–Vis absorption spectrometer in an electrochromic absorption band shift spectra experiment.

**Table S1.** log*P* of [C_n_mim]Br (Fan et al.,2016)

|  | log*P* |
| --- | --- |
| [C_4_mim]Br | − 2.00 |
| [C_6_mim]Br | − 0.91 |
| [C_8_mim]Br | 0.25 |
| [C_10_mim]Br | 1.4 |


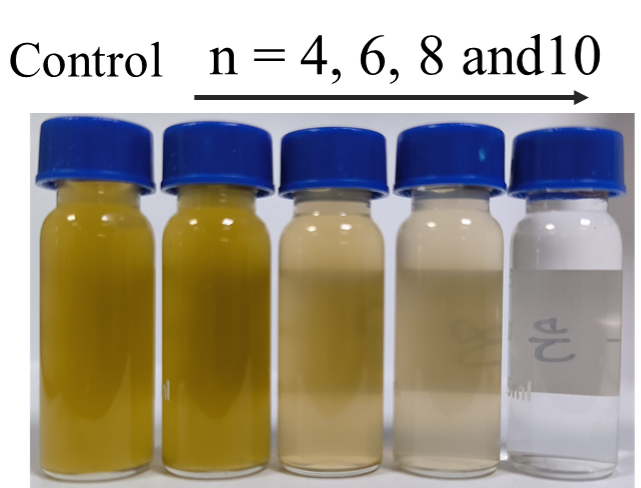


**Supplementary Figure S2** Bacterial growth status exposed in 2 mM [C_n_mim]Br


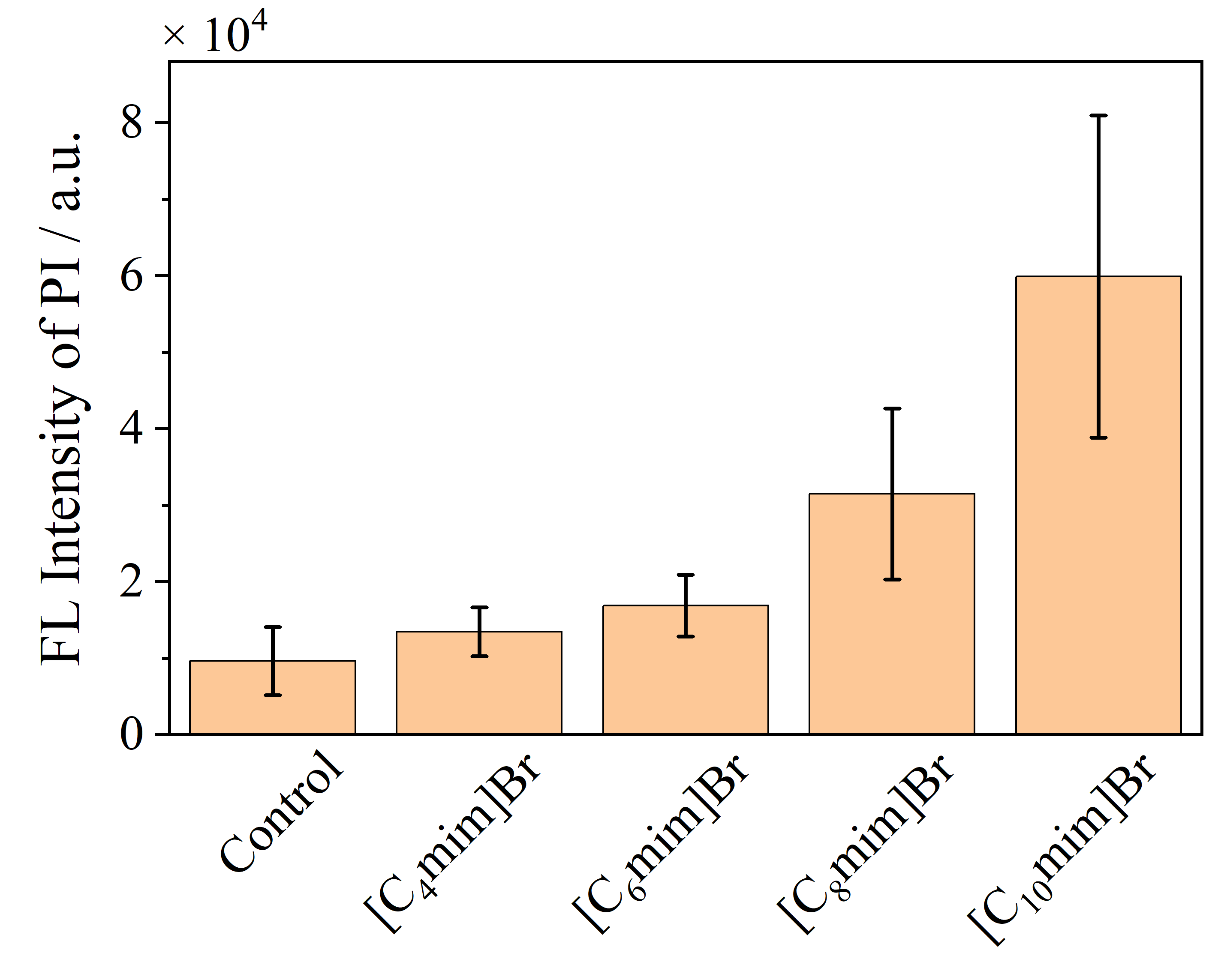


**Supplementary Figure S3** Fluorescence intensity of propidium iodide in *R. sph* incubation with 5 mM [C_n_mim]Br for 4 h.


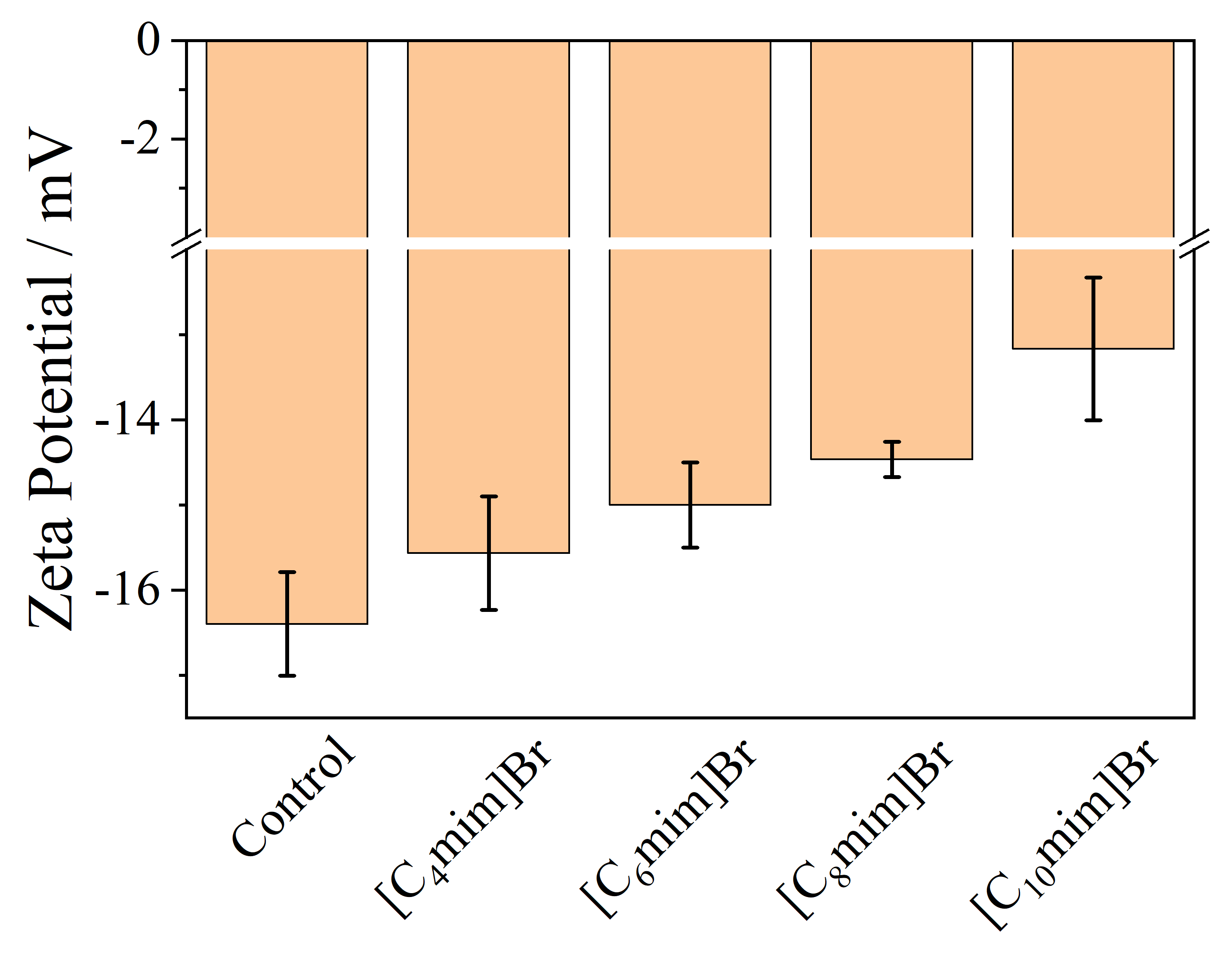


**Supplementary Figure S4** Zeta potential of *R. sph* incubation with 5 mM [C_n_mim]Br for 4 h.


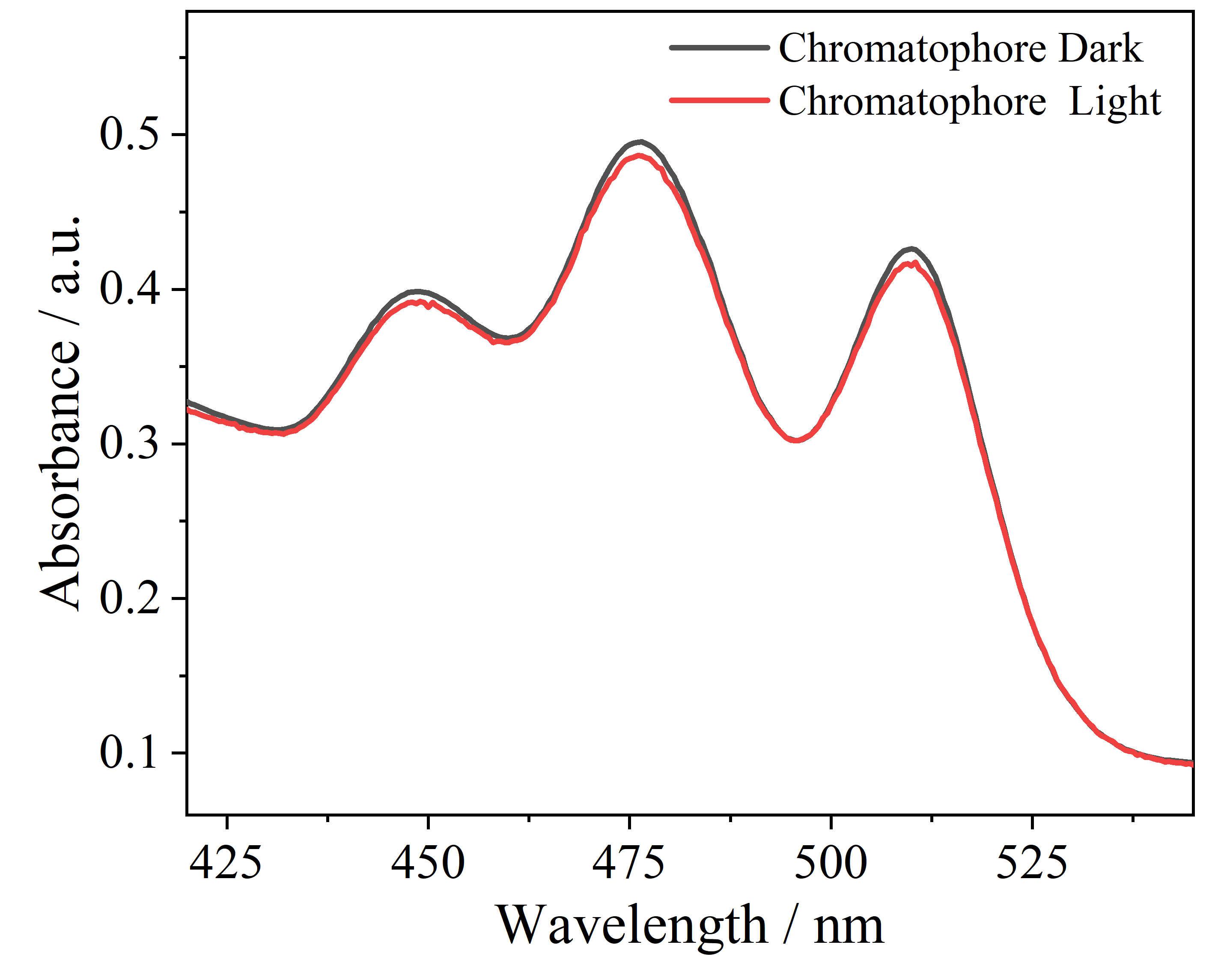


**Supplementary Figure S5** Uv-vis absorption spectrum of carotenoid in chromatophores from *R. sph* under light and dark.

**Table S2.** ΔOD_amp_ of chromatophore without or with 5 mM ILs. ΔOD_amp_ = ΔOD_526_ − ΔOD_510_

|  | Peak site  (Amplitude) | | Peak site  (Amplitude) | ΔOD_amp_ |
| --- | --- | --- | --- | --- |
| Control | | 525  (0.0026) | 510  (− 0.0078) | 0.0105 |
| [C_4_mim] Br | | 527  (0.0020) | 510  (− 0.0061) | 0.0081 |
| [C_6_mim] Br | | 528  (0.0014) | 510  (− 0.0053) | 0.0067 |
| [C_8_mim] Br | | 536  (0.0009) | 514  (− 0.0048) | 0.0057 |
| [C_10_mim] Br | | 539  (0.0011) | 514  (− 0.0037) | 0.0048 |

**Reference**

Fan, Y., Dong, X., Yan, L., Li, D., Hua, S., and Hu, C. (2016). Evaluation of the toxicity of ionic liquids on trypsin: A mechanism study. *Chemosphere*, 148, 241-247. https://doi.org/10.1016/j.chemosphere.2016.01.033
